# Spotlight on the Artifacts in Next-Generation Sequencing in PGT-A: Reason for High Mosaicism Reporting

**DOI:** 10.5935/1518-0557.20250018

**Published:** 2025

**Authors:** Neeta Singh, Ankita Sethi, Ritu Gupta, Lata Rani, Monika Saini

**Affiliations:** 1 Department of Obstetrics and Gynecology, All India Institute of Medical Sciences (AIIMS), New Delhi, India; 2 Laboratory Oncology Unit, Dr.B.R.A. IRCH, All India Institute of Medical Sciences (AIIMS), New Delhi; 3 Genomics Lab, Centralized Core Research Facility (CCRF), All India Institute of Medical Sciences (AIIMS), New Delhi, India

**Keywords:** next-generation sequencing, PGT-A, mosaicism, artifacts

## Abstract

The study investigates artifacts in Next-Generation Sequencing (NGS) used for Preimplantation Genetic Testing for Aneuploidy (PGT-A) and their contribution to the high rates of mosaicism reporting. Modern PGT-A can detect mosaicism by analyzing copy number variations (CNVs) in embryonic biopsies, yet distinguishing true mosaicism from artifacts remains challenging. In a cohort of 22 embryos, NGS profiles revealed recurring artifacts on chromosomes 7, 11, 16, and 19. These artifacts likely result from errors in DNA amplification for NGS library preparation, potentially leading to false mosaicism diagnosis. The study utilized DNA extracted from trophectoderm biopsies, spent culture media, and whole blastocyst samples, with CNV analysis performed using BlueFuse Multi software. Quality control parameters such as DLR noise, read count, and quality score were considered, confirming that technical inconsistencies contribute to the observed artifacts. Findings align with prior research, suggesting the need for improved NGS protocols to minimize these errors. Enhanced internal validation and adoption of new technologies could reduce false-positive rates and improve clinical decision-making in PGT-A.

## INTRODUCTION

Advances in Next Generation Sequencing (NGS) over the past decade have significantly increased the use of Preimplantation Genetic Testing for Aneuploidy (PGT-A) in Assisted Reproductive Technology (ART) clinics, particularly for women of advanced maternal age. However, a key challenge remains in distinguishing genuine pathological copy number variations (CNVs) in embryonic NGS profiles from artifacts caused by errors in DNA amplification or library preparation (Morales, 2024). Chromosomal mosaicism, where cells with different chromosomal contents coexist, has been recognized in human embryos for over three decades. Earlier versions of PGT-A were unable to detect mosaicism-either because only a single cell was tested or the technology lacked the precision to identify it. As a result, embryos were classified simply as normal or abnormal, which likely led to misdiagnoses and adverse clinical outcomes. Modern PGT-A now uses multicellular biopsies and advanced technologies that can detect intermediate copy number variations across whole chromosomes or specific regions, making the issue of mosaicism unavoidable and presenting new challenges in clinical decision-making for embryos with such findings (Popovic *et al.*, 2020; Viotti, 2020; Viotti *et al.*, 2021).

The data available with us (n=22 embryos) was examined to assess whether technical errors inherent to Next-Generation Sequencing could potentially introduce artifacts, thereby erroneously increasing the reported incidence of Chromosomal Mosaicism.

### Study design

The data examined is from a prospective cohort study conducted at the ART Centre, Department of Obstetrics and Gynaecology, in collaboration with the Genomics Lab, Centralized Core Research Facility (CCRF) at AIIMS, New Delhi. All participants provided written informed consent to undergo preimplantation genetic testing for aneuploidy (PGT-A), and the study received ethical approval from the Institute Ethics Committee. Patients included in the study met the following criteria: age≥ 35 years, experienced one or more implantation failures, severe male factor infertility, and opted for elective single euploid blastocyst transfer (eSET). In cases where embryos were found to be aneuploid following trophectoderm (TE) biopsy during PGT-A, embryos were donated for research purpose in the year 2022.

## METHODS

The DNA extraction and amplification was carried out from TE biopsy and SCM using the Sureplex DNA amplification system. The libraries were prepared using VeriSeqTM PGS Library Preparation kit. A total of 24 libraries were pooled, denatured and subjected to NGS using Illumina MiSeq system. CNV visualization and analysis for each sample was carried out using BlueFuse Multi Software (Illumina, USA).The aneuploid whole blastocyst were subjected to the same protocol.

### Data analysis

The FASTQ and BAM files were generated in the MiSeq system and subjected to Blufuse Multi software (Illumina) for data visualization and CNV analysis. GRCh37 was used as the reference genome and the threshold for aneuploidies was set based on the size of the CNV change ≥20Mb.

Since the Blufuse Multi software has limitations for detection of mosaicism, it was detected manually with the threshold levels (20-80% aneuploid cells).

In this study, embryos/ SCM with <20% aneuploidy was considered euploid while >80% was considered aneuploid, 20-50% was considered as low mosaic and 51-80% was considered high mosaic, embryos having aneuploidy as well as mosaicism in any of the chromosome were considered aneuploid-mosaic. The quality control parameters considered for successful sequencing are the DLR Noise (≤0.4), the number of reads after filtering (2,50,000) and the average quality score (>30).

## RESULTS

**Figure 1:**
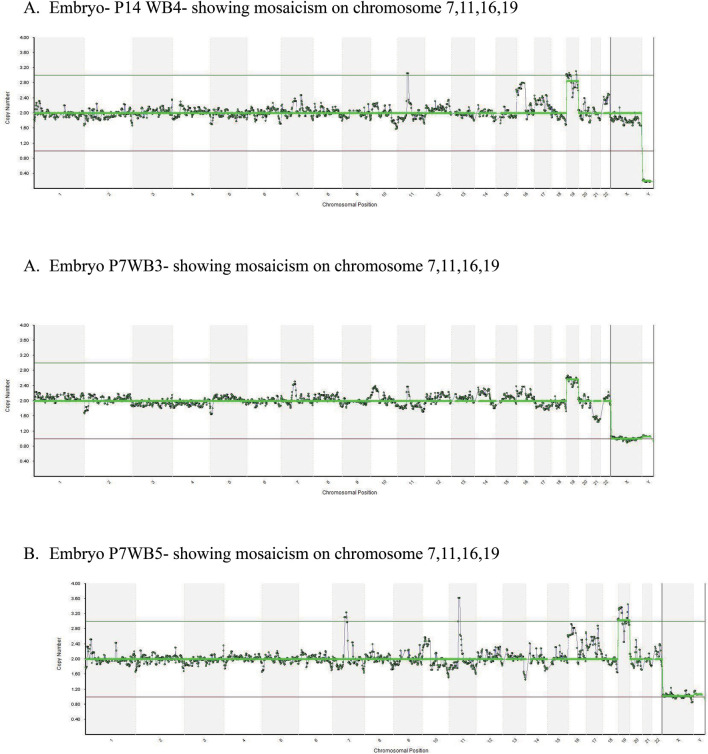
CNV charts of the whole blastocyst NGS analysis showing mosaicism on chromosome 7,11,16,19.

NGS profiles generated from the aneuploid whole blastocysts, TE biopsy and SCM, were manually analyzed for copy number variations and for any common genomic artifacts. The quality control was performed using Illumina Quality Control requirements and only samples satisfying these requirements were used for analysis in the study. Common artifacts were identified affecting chromosomes 7,11,16 and 19. Out of 22 embryos, NGS profile of 21 blastocysts had artifacts at chromosome 19, and 15 had artifacts at chromosome 7,11,16. Figure 1 shows the CNV charts of the whole blastocyst NGS analysis showing mosaicism on chromosome 7,11,16,19. These artifacts may be introduced during whole genome amplification and/or suboptimal NGS library preparation or are an inherent weakness of the NGS library preparation kit. There are newer NGS profiling kits available with advanced technology, which may overcome these technical errors and decrease these artifacts. Table 1 shows quality control parameters for NGS library preparation and sequencing.

**Table 1:** Quality control parameters for library preparation and sequencing.

	TE Biopsy (Mean±SD)	SCM (Mean±SD)	WB (Mean±SD)
**DLR noise**	0.21±0.040	0.24±0.05	0.22±0.041
**Number of Reads after Filtering**	514429.2±220337.5	533565.1±171768.3	514002.2±220447.5
**Average Quality Score**	35.38±0.23	35.34±0.20	35.48±0.20

Mosaicism refers to the presence of two or more distinct cell lines within an embryo, resulting from post-zygotic errors. Artifacts, on the other hand, are technical errors or anomalies introduced during sample collection, processing, or analysis.

### Key Differentiation Methods

**Signal Consistency Across Reads or Regions:** In mosaicism, the chromosomal abnormalities appear consistently across multiple reads or regions of analysis, reflecting a biological event. Artifacts, however, tend to present as random, sporadic errors without consistent patterns.

**Next-Generation Sequencing (NGS):** Mosaicism exhibits distinct intermediate copy number variations (e.g., 30-70% abnormal cells) in the read depth, while artifacts often show irregular spikes or drops that do not match biological variability.

**Predefined Mosaicism Thresholds:** We used established thresholds to categorize mosaicism, such as detecting abnormalities in 20-80% of cells. Deviations below 20% or above 80% are more likely to result from artifacts or noise, rather than true mosaicism.

### Quality Control in Laboratory Processes

Stringent quality control measures were implemented, including avoiding DNA contamination during biopsy and amplification, optimizing sample preparation to minimize sequencing errors and PCR noise. The QA and QC of NGS analysis was up to the mark.

The consistent observation of the same pattern of mosaicism in chromosomes 7, 11, 16, and 19 across whole embryo NGS analysis suggests the possibility of a technical artifact. Due to budget constraints validation through repeat analysis, to confirm mosaic findings and rule out artifacts, could not be performed.

## DISCUSSION

The occurrence of artifacts on specific chromosomes, such as chromosomes 7, 11, 16, and 19, indicates that these may be due to technical errors related to whole genome amplification or library preparation, rather than genuine embryonic mosaicism. Laboratories with limited experience in NGS may mistakenly interpret these artifacts as mosaicism, leading to potential over-diagnosis. To minimize false-positive results in reporting mosaicism, especially in the context of IVF and PGT-A, it is essential to perform thorough internal validation and implement rigorous quality control measures. The development of newer NGS kits with more advanced technologies could potentially address these issues, thereby improving accuracy in detecting mosaicism.

Tilley *et al.* (2022) analyzed NGS profiles from biopsied human embryonic trophectoderm cells and identified common genomic artifacts affecting chromosomes 7, 11, and 19. These artifacts mimicked small CNVs and were associated with increased sequence counts at the centromeres of chromosomes 7 and 11 and across chromosome 19. The study found that repeating the library preparation and sequencing normalized these artifacts, suggesting that technical issues during library preparation may introduce them. Awareness of such artifacts can help reduce false positives or inconclusive results, thereby enhancing the clinical use of embryos after PGT-A (Tilley *et al.*, 2022). Our findings are consistent with this study, with the additional observation of artifacts on chromosome 16.

Gigg & Paulson (2024) reported that PGT-A is now performed in nearly half of all IVF cycles in the U.S. Since its validation in 2014, NGS has become the standard method for PGT-A, sequencing the DNA of biopsied trophectoderm cells. The percentage of abnormal DNA determines whether the embryo is classified as euploid, aneuploid, or mosaic. Intermediate copy number (ICN) can be attributed to mosaicism, mitotic state, biopsy technique, amplification bias, or statistical noise. Different commercial PGT providers (CPPs) use varying ICN thresholds to report mosaicism, resulting in reported rates ranging from 2.6% to 17.7%. Gigg & Paulson (2024) found that mosaicism rates were influenced by the ICN criteria, with broader thresholds leading to higher reported rates. Lower mosaic rates may seem favorable, but stricter ICN criteria could result in a larger number of embryos being classified as aneuploid. They emphasize the need for standardized ICN criteria to harmonize reporting of overall mosaicism and distinguish between lowand high-level mosaics (Gigg & Paulson, 2024).

Chen *et al.* (2024) explored artifacts in NGS in cancer patients, specifically comparing sonication and enzymatic fragmentation during DNA library preparation. They found that enzymatic fragmentation resulted in more artifacts than sonication, with distinct chimeric artifact reads appearing in both methods. Based on their findings, they proposed a mechanistic model, PDSM (pairing of partial single strands derived from a similar molecule), to explain these errors. They also developed a bioinformatic algorithm to filter out these sequencing artifacts. Similarly, NGS used in PGT-A should adopt bioinformatic tools to generate a custom artifact list to avoid overdiagnosis of mosaicism (Chen *et al.*, 2024).

## CONCLUSION

Four common artifacts were identified on chromosomes 7, 11, 16, and 19, likely introduced during WGA or NGS library preparation. Awareness of these artifacts is essential for reducing false-positive mosaicism rates and improving clinical outcomes in PGT-A. Correcting technical errors through repeated WGA or better NGS profiling kits may increase the chances of clinical embryo utilization, particularly for advanced-age women, where accurate embryo selection is critical for successful ART cycles.
